# Supplementary material for: Novel Driver Strength Index highlights important cancer genes in TCGA PanCanAtlas patients
Source: PeerJ. 2022 Aug 11;10:e13860. doi: 10.7717/peerj.13860 (PMC9375969; doi:10.7717/peerj.13860)
Supplement: Supplemental Information 2 [file peerj-10-13860-s002.zip › COHORTS/genes plots/cohorts/2021_8_16_14_9_distribution_cohorts_males_KIRP.pdf]

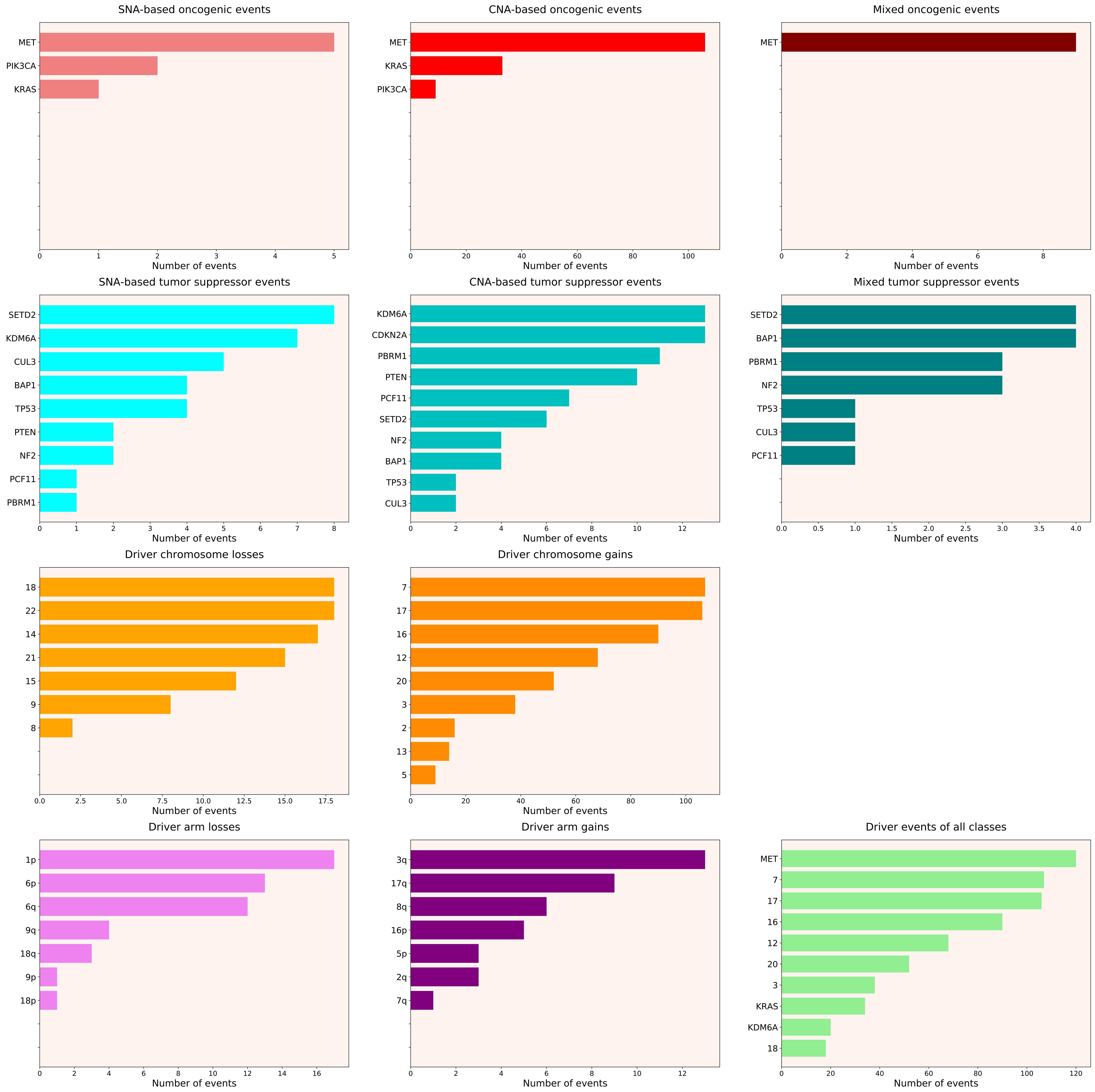
SNA-based oncogenic events
MET
PIK3CA
KRAS
0 1 2 3 4 5
Number of events
CNA-based oncogenic events
MET
KRAS
PIK3CA
0 20 40 60 80 100
Number of events
Mixed oncogenic events
MET
0 2 4 6 8
Number of events
SNA-based tumor suppressor events
SETD2
KDM6A
CUL3
BAP1
TP53
PTEN
NF2
PCF11
PBRM1
0 1 2 3 4 5 6 7 8
Number of events
CNA-based tumor suppressor events
KDM6A
CDKN2A
PBRM1
PTEN
PCF11
SETD2
NF2
BAP1
TP53
CUL3
0 2 4 6 8 10 12
Number of events
Mixed tumor suppressor events
SETD2
BAP1
PBRM1
NF2
TP53
CUL3
PCF11
0.0 0.5 1.0 1.5 2.0 2.5 3.0 3.5 4.0
Number of events
Driver chromosome losses
18
22
14
21
15
9
8
0.0 2.5 5.0 7.5 10.0 12.5 15.0 17.5
Number of events
Driver chromosome gains
7
17
16
12
20
3
2
13
5
0 20 40 60 80 100
Number of events
Driver arm losses
1p
6p
6q
9q
18q
9p
18p
0 2 4 6 8 10 12 14 16
Number of events
Driver arm gains
3q
17q
8q
16p
5p
2q
7q
0 2 4 6 8 10 12
Number of events
Driver events of all classes
MET
7
17
16
12
20
3
KRAS
KDM6A
18
0 20 40 60 80 100 120
Number of events
